# Adaptation of *Scheffersomyces stipitis* to hardwood spent sulfite liquor by evolutionary engineering

**DOI:** 10.1186/s13068-015-0234-y

**Published:** 2015-03-26

**Authors:** Susana R Pereira, Violeta Sànchez i Nogué, Cláudio J R Frazão, Luísa S Serafim, Marie F Gorwa-Grauslund, Ana M R B Xavier

**Affiliations:** CICECO - Aveiro Institute of Materials and Departamento de Química, Universidade de Aveiro, Campus Universitário de Santiago, Aveiro, 3810-193 Portugal; Division of Applied Microbiology, Department of Chemistry, Lund University, PO Box 124, Lund, Sweden

**Keywords:** Hardwood spent sulfite liquor (HSSL), *Scheffersomyces stipitis*, Lignocellulosic inhibitors, Evolutionary engineering, Bioethanol

## Abstract

**Background:**

Hardwood spent sulfite liquor (HSSL) is a by-product of acid sulfite pulping process that is rich in xylose, a monosaccharide that can be fermented to ethanol by *Scheffersomyces stipitis*. However, HSSL also contains acetic acid and lignosulfonates that are inhibitory compounds of yeast growth. The main objective of this study was the use of an evolutionary engineering strategy to obtain variants of *S. stipitis* with increased tolerance to HSSL inhibitors while maintaining the ability to ferment xylose to ethanol.

**Results:**

A continuous reactor with gradually increasing HSSL concentrations, from 20% to 60% (*v*/*v*), was operated for 382 generations. From the final obtained population (POP), a stable clone (C_4_) was isolated and characterized in 60% undetoxified HSSL. C_4_ isolate was then compared with both the parental strain (PAR) and POP. Both POP and C_4_ were able to grow in 60% undetoxified HSSL, with a higher capability to withstand HSSL inhibitors than PAR. Higher substrate uptake rates, 7% higher ethanol efficiency and improved ethanol yield were obtained using C4.

**Conclusion:**

*S. stipitis* was successfully adapted to 60% (*v*/*v*) undetoxified eucalyptus HSSL. A stable isolate, C_4_, with an improved performance in undetoxified HSSL compared to PAR was successfully obtained from POP. Owing to its improved tolerance to inhibitors, C_4_ may represent a major advantage for the production of bioethanol using HSSL as substrate.

## Background

Over the last two decades, second generation (2G) bioethanol, that is bioethanol produced from lignocellulosic feedstock, has received increasing attention worldwide. Tested feedstocks include lignocellulosic forest or agricultural residues and industrial by-products [1-3]. But spent sulfite liquors (SSLs) that are by-products from pulp and paper industry produced in large scale can also be considered promising for 2G bioethanol production [4]. SSL is the main effluent resulting from the acid sulfite-pulping process of wood [5] and contains significant amounts of dissolved organic matter (COD > 100,000 mgO_2_.L^−1^) [4]. SSL is normally concentrated by evaporation and then burned for energy generation and base recovering [5].

The potential of SSL from hardwood (HSSL) as raw material for bioprocesses has not been fully exploited yet [4], mostly, because HSSL is rich in pentose sugars whose fermentation into bioethanol is considered a challenge for a majority of microorganisms [4,6]. In that context, *Scheffersomyces stipitis* could be an interesting biocatalyst as it is one of the most efficient yeasts to naturally ferment pentoses to ethanol under appropriate oxygenation conditions [7]. However, HSSL also contains significant amounts of acetic acid (8 to 11 g.L^−1^) and lignosulfonates (60 to 80 g.L^−1^), known microbial inhibitors that limit the possibility of its bioprocessing [4,8,9]. Xavier *et al*. indeed observed that *S. stipitis* NRRL Y-7124 could not grow in medium containing over 40% (*v*/*v*) eucalyptus-based HSSL without a previous detoxification process [8]. From the tested HSSL bio-detoxification methods, the use of the filamentous fungus *Paecilomyces variotii* NRRL-1115 provided the more sustainable process to obtain a suitable substrate for further *S. stipitis* growth and fermentation [10]. However, during the fungal bio-detoxification step, only part of the inhibitors was removed, which resulted in a final ethanol yield lower than the one attained using HSSL detoxified by ion exchange resins, a more expensive option [8].

It is well known that yeasts, such as *Saccharomyces cerevisiae*, can adapt gradually to tolerate and grow in the presence of inhibitors, or other harsh environmental conditions [11-13]. In addition, evolutionary engineering strategies have been successfully applied to *S. cerevisiae* for optimizing bioethanol production from lignocellulosic materials, by improving, for instance, simultaneous fermentation of xylose and arabinose [14], by enhancing the tolerance to high temperature and inhibitors [15] and by improving the tolerance to hydrolysates of lignocellulosic biomass [16]. In contrast, only limited attempts to adapt *S. stipitis* to typical inhibitors present in SSLs have been reported [4]. Mohandas *et al*. adapted *S. stipitis* to high acetic acid concentrations using shake flasks cultures with increasing acid levels [17]. The obtained mutant showed a shorter fermentation time, a higher ethanol yield and tolerance to acetic acid in wood hydrolysates at lower pH [17]. Using also shake flasks cultures, Nigam was able to obtain a mutant of *S. stipitis* adapted to an acidic hydrolysate of hardwood hemicellulose with improved growth and providing higher ethanol yield [18]. Later, this approach was used to adapt *S. stipitis* to a red oak HSSL, improving the ethanol fermentation yield [19]. Using random mutagenesis, Bajwa *et al*. obtained mutants of *S. stipitis* with enhanced tolerance to HSSL inhibitors and the capacity to generate higher ethanol yields than the parental strain [20]. Another mutant strain, obtained by Hughes *et al*. using UV mutagenesis [21], was able to grow anaerobically on xylose/glucose substrate and showed a higher ethanol production than an industrial strain of *S. cerevisiae* [21]. Therefore, random mutation plus a natural selection of strains can be a good alternative to the classical genomic approaches, in order to obtain more robust yeasts. These methods are particularly useful since they provide resistant yeasts to multiple stress factors [12,22].

The studies described were performed either in shake flasks, that is under conditions where oxygenation, pH and growth state are undefined, or through mutagenesis, where many additional unwanted mutations can be generated. Therefore, the main purpose of the present study was to improve *S. stipitis* tolerance towards undetoxified *Eucalyptus globulus* HSSL by long-term fermentation in a continuous stirred tank reactor (CSTR) with controlled conditions. The CSTR was operated for 382 generations with increasing HSSL concentrations, from 20% to 60% (*v*/*v*). From the obtained final population in the CSTR (POP), ten isolates were randomly selected from the fastest grown colonies and characterized in the presence of 60% (*v*/*v*) of undetoxified HSSL. The most efficient isolate was selected and characterized, together with both POP and the parental strain (PAR), in a batch reactor with 60% (*v*/*v*) undetoxified HSSL.

## Results

### Continuous adaptation of *S. stipitis* to HSSL

**Figure 1:**
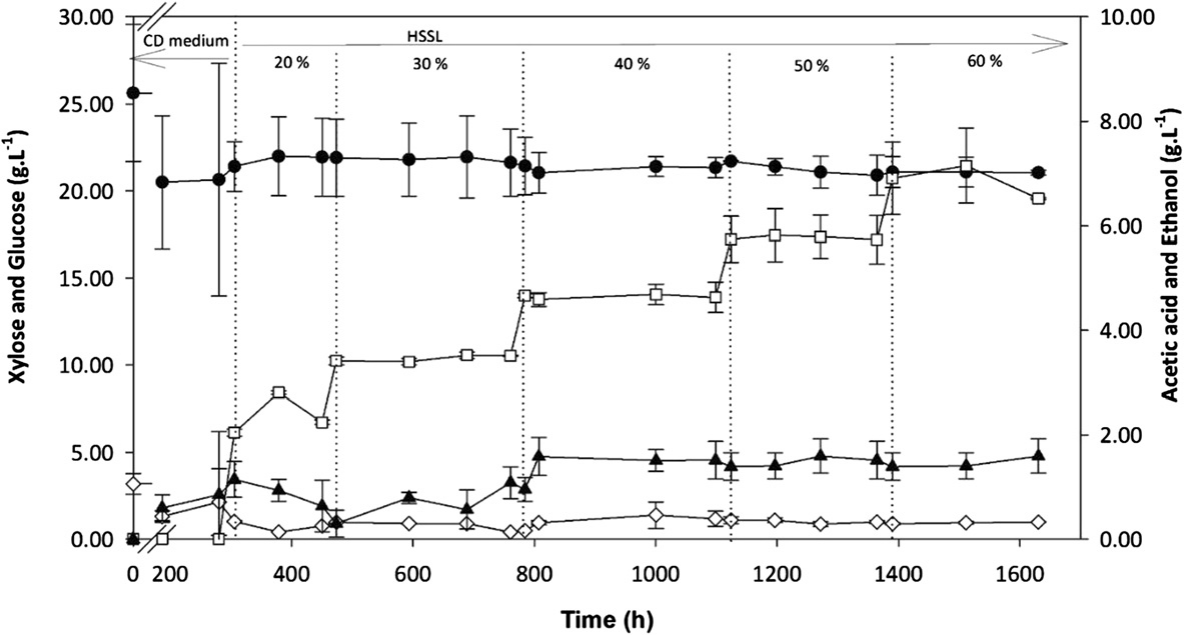
**Substrate consumption and ethanol production over time during adaptation of** ***S. stipitis*** **NRRL-7124 to increasing concentrations of undetoxified HSSL, from 20% to 60% (** ***v*** **/** ***v*** **), in a CSTR.** The points correspond to mean values of two different analyses. Xylose (black circle), glucose (white diamond), acetic acid (white square) and ethanol (black up-pointing triangle).

A continuous stirred tank reactor (CSTR) with the objective to adapt *S. stipitis* to higher concentrations of inhibitors was operated with increasing undetoxified HSSL concentrations (Figure 1), while maintaining a sugar concentration corresponding to undiluted HSSL (25 g.L^−1^ xylose and 2.3 g.L^−1^ glucose). Initially, the CSTR worked for 280 h with chemically defined (CD) medium, and during this period, xylose was consumed at a rate of 1.0 g.L^−1^.h^−1^. When HSSL started to be pumped into the reactor, xylose consumption decreased to 0.74 g.L^−1^.h^−1^. Similarly, ethanol volumetric production rate also decreased from 0.17 to 0.13 g.L^−1^.h^−1^. However, along the several increments in HSSL concentration (from 20% to 60%), no substantial variation in the substrate consumption was further observed (Figure 1). The consumption of xylose was 0.85 ± 0.08 g.L^−1^.h^−1^ and of glucose was 0.46 ± 0.06 g.L^−1^.h^−1^. No consumption of acetic acid was observed along the CSTR operation. In contrast to sugars, ethanol production rate increased to 0.30 g.L^−1^.h^−1^ with 30% HSSL, stabilizing through the subsequent HSSL increments (from 30% to 60%) and averaging 0.31 ± 0.01 g.L^−1^.h^−1^. The CSTR was ended after 382 generations when the culture reached a steady state with 60% (*v*/*v*) of undetoxified HSSL.

### Isolation and evaluation of isolates in shake flasks

**Figure 2:**
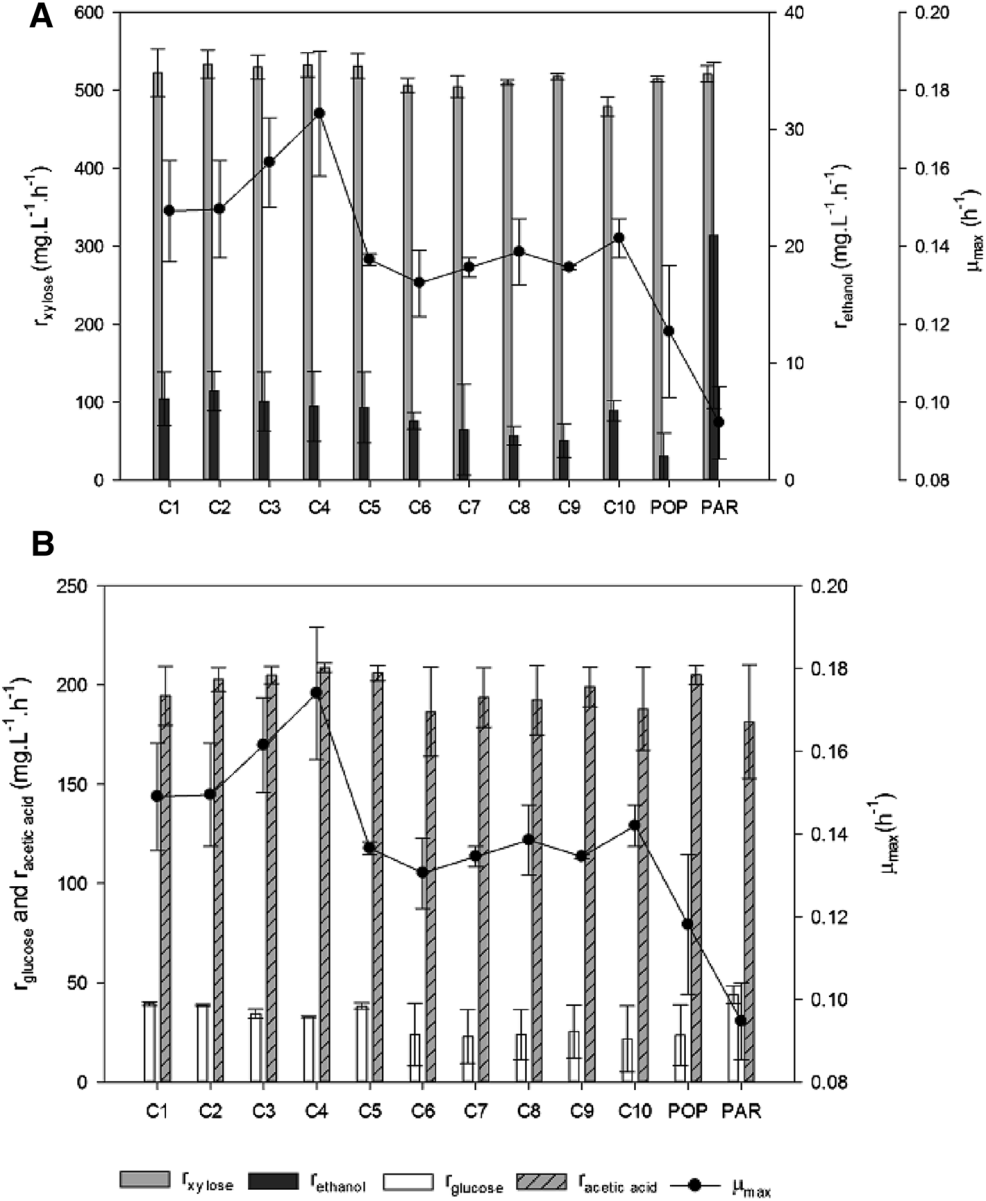
**Calculated kinetic parameters for the ten isolates, PAR and POP strains grown in shake flasks containing 60% undetoxified HSSL.** The values represent means of two biological replicas. **(A)** *r*_xylose_ (grey bars), *r*_ethanol_ (black bars) (mg.L^−1^.h^−1^) and *μ*_max_ (black dots) (h^−1^); **(B)** *r*_glucose_ (white bars), *r*_acetic acid_ (stripes) (mg.L^−1^.h^−1^) and *μ*_max_ (black dots) (h^−1^). PAR, parental strain; POP, population.

The final population obtained in the CSTR (named POP) was plated into solid YM_xyl_ medium containing 60% (*v*/*v*) undetoxified HSSL and the first ten isolated colonies to grow were selected. In order to verify the stability of the acquired phenotype for the ten adapted isolates (C_1_ to C_10_), each one was plated for ten sequential transfers under non-selective conditions (YM_xyl_ plates without any HSSL). After the plating sequence, the ten isolates were tested in liquid cultures of CD medium with 60% (*v*/*v*) undetoxified HSSL and compared to PAR and POP for maximum specific growth rate, xylose, glucose and acetic acid uptake rates and ethanol production rate (Figure 2).

POP achieved a higher *μ*_max_ (0.118 h^−1^) than PAR (0.095 h^−1^). The *μ*_max_ attained by the different isolates was generally similar to the one achieved by POP. Amongst the isolates, C_4_ displayed the highest *μ*_max_ (0.174 h^−1^) and also the highest xylose uptake rate (0.532 g.L^−1^.h^−1^). C_4_ was also amongst the five isolates (C_1_ to C_5_) that attained the highest glucose consumption rates with an average of 38.5 ± 2.5 mg.L^−1^.h^−1^ presenting 32.8 mg.L^−1^.h^−1^ (Figure 2). Finally, C_4_ was amongst the isolates the one with the highest acetic acid uptake rate (209 mg.L^−1^.h^−1^) (Figure 2).

Ethanol production rate was lower in POP (2.01 mg.L^−1^.h^−1^) than in PAR (20.9 mg.L^−1^.h^−1^), and all isolates displayed intermediate level between POP and PAR, with an average production rate of 5.63 ± 1.19 mg.L^−1^.h^−1^ (Figure 2). The lowest ethanol production rate was observed in an experiment with C_9_, 3.38 mg.L^−1^.h^−1^, and the highest rate was achieved by C_2_, 7.62 mg.L^−1^.h^−1^. Nevertheless, C_4_ attained an ethanol production rate of 6.30 mg.L^−1^.h^−1^, closer to the one achieved by C_2_ (Figure 2). For all these facts, C_4_ was chosen to be further studied.

### Comparison of PAR, POP and C_4_ in bioreactor

**Figure 3:**
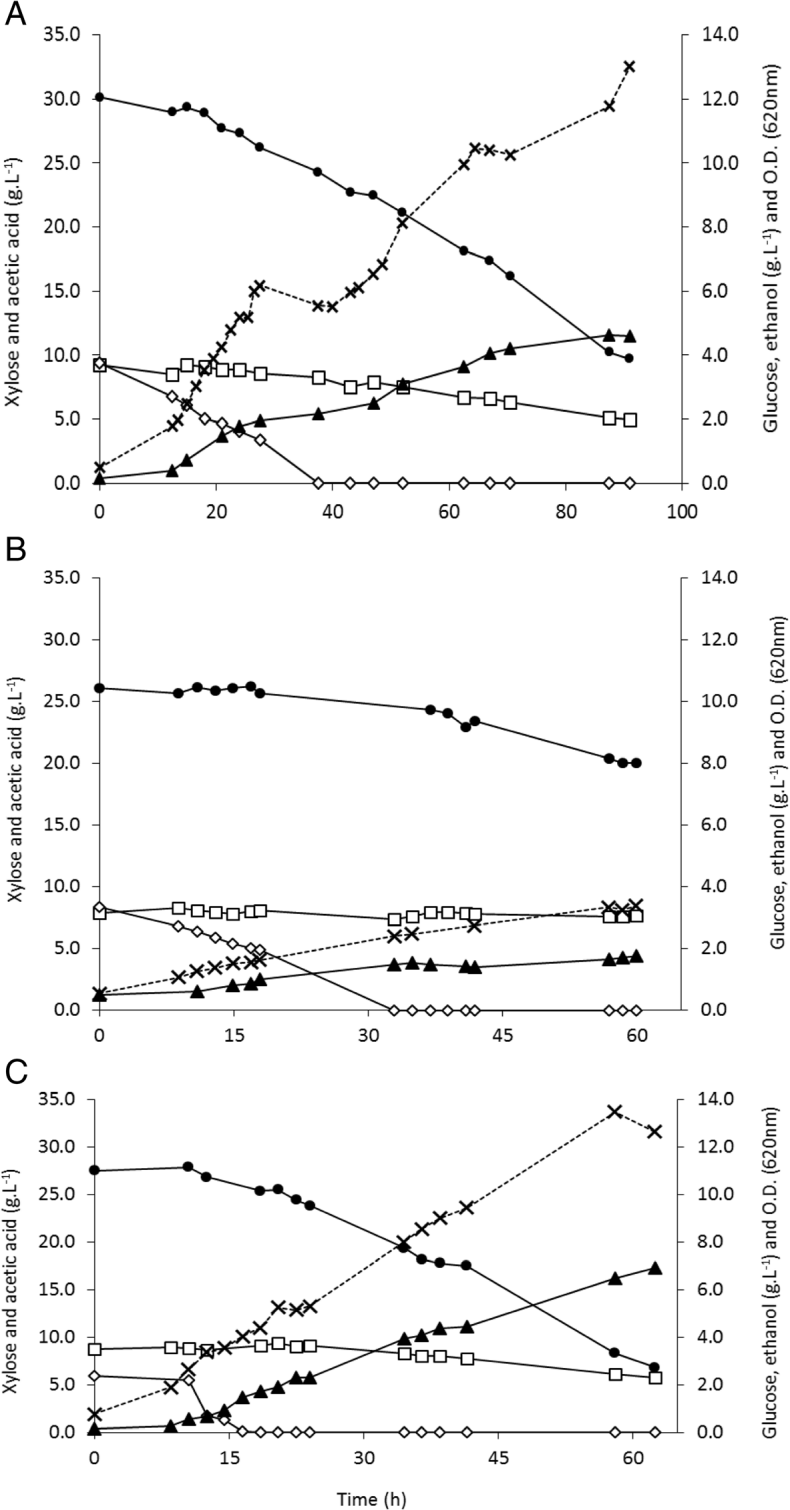
**Substrates consumption and ethanol production over time during fermentation in 60% undetoxified HSSL bioreactors of C**_**4**_ **(A), PAR (B) and POP (C).** The points represented in each graph refer to the values obtained in single experiments. Xylose (black circle), glucose (white diamond), acetic acid (white square), ethanol (black up-pointing triangle) and O.D.620_nm_ (multiplication sign).

Table 1 shows the kinetic and stoichiometric parameters obtained in batch bioreactors by PAR, POP and C_4_. All the tests were performed until the stationary phase was reached and, consequently, the duration of each batch was different. PAR and POP gave a similar batch duration, 61 and 62 h, respectively (Figure 3B,C), whereas C_4_ needed 91 h (Figure 3A) to reach the stationary phase. Major differences in the kinetic parameters were also observed between PAR, POP and C_4_ (Table 1). Despite similar *μ*_max_ (0.037 h^−1^) for POP and PAR, the final biomass concentration achieved by POP was 2.6 higher (3.75 g.L^−1^) than the one attained by PAR (1.45 g.L^−1^) (Table 1). However, C_4_ achieved the highest *μ*_max_ (0.067 h^−1^) but the final biomass (3.57 g.L^−1^) was similar to POP. Although C_4_ had the highest *μ*_max_, the deceleration phase was longer than the one showed by POP so C_4_ ended up with a longer fermentation time and both cultures achieved a similar final biomass value. The colony-forming units (CFU) obtained from POP (3.5 × 10^8^ cells.mL^−1^) and C_4_ (2.4 × 10^8^ cells.mL^−1^) were four and three times higher, respectively, than those from PAR (7.8 × 10^7^ cells.mL^−1^).

**Table 1:** **Kinetic and stoichiometric parameters of** ***S. stipitis*** **PAR, POP and C**_**4**_ **in bioreactor cultivations with 60% non-detoxified HSSL**

**Parameters**	**PAR**	**POP**	**C**_**4**_
*μ*_max_ (h^−1^)	0.037	0.037	0.067
Lag phase (h)	12	10	10
Batch duration (h)	61	62	91
Final biomass (g.L^−1^)	1.45 ± 0.08	3.75 ± 0.14	3.57 ± 0.13
Final CFU (cells.mL^−1^)	7.8 × 10^7^ ± 6.0 × 10^6^	3.5 × 10^8^ ± 3.0 × 10^7^	2.4 × 10^8^ ± 5.8 × 10^7^
Final xylose consumed (g.L^−1^)	6.1	20.7	20.4
*r*_xylose_ (g.L^−1^.h^−1^)	0.10	0.33	0.22
*r*_glucose_ (g.L^−1^.h^−1^)	0.10	0.13	0.10
*r*_acetic acid_ (g.L^−1^.h^−1^)	0.003	0.050	0.050
*r*_ethanol_ (g.L^−1^.h^−1^)	0.02	0.11	0.05
[EtOH]_max_ (g.L^−1^)	1.76	6.93	4.60
*Y*_ethanol_ (g.g^−1^)^a^	0.13	0.26	0.16
Conversion efficiency (%)^a^	26	51	32

^a^Calculated at the maximum ethanol concentration. *r* refers to consumption rates for xylose, glucose and acetic acid and production rate for ethanol. PAR, parental strain; POP, population.

The substrate consumption rates were lower in PAR fermentation (Table 1). POP attained the highest xylose uptake rate (0.33 g.L^−1^.h^−1^). C_4_ achieved a lower xylose consumption rate (0.22 g.L^−1^.h^−1^) than POP but still higher than the one attained by PAR (0.10 g.L^−1^.h^−1^). Moreover, the amount of xylose consumed by POP and C_4_ was similar, 20.7 and 20.4 g.L^−1^, respectively. On the contrary, PAR consumed less than a third of the xylose consumed by POP or C_4_, only 6.1 g.L^−1^. The acetic acid uptake rate was similar for POP and C_4_ (0.05 g.L^−1^.h^−1^), while PAR presented a lower rate. The glucose uptake rate was similar in all trials (Table 1). Considering the maximum ethanol concentration, PAR attained the lowest value (1.76 g.L^−1^), while POP reached the highest with 6.93 g.L^−1^. The ethanol conversion efficiency was 51% for POP, 32% for C_4_ and only 26% for PAR.

## Discussion

Evolutionary engineering was used to obtain a stable isolate of *S. stipitis* NRRLY-7124 strain that would tolerate the inhibitory compounds present in HSSL, thereby enabling growth and bioethanol production without preliminary detoxification step. By increasing HSSL concentrations in a CSTR, a HSSL-tolerant population was obtained, from which the promising isolate C_4_ was selected. C_4_ was able to better withstand undetoxified 60% HSSL, with improved sugar and acetic acid uptake and increased biomass and ethanol production as compared to the original strain (PAR). And although its performances were slightly below those of the population from which it was originated (POP), C_4_ was a stable tolerant strain in contrast to POP.

All the POP isolates that were analysed presented distinct behaviour in terms of sugar and acetic acid uptake rates, biomass and ethanol production, indicating that POP was a heterogeneous population of *S. stipitis*. According to Holland *et al*. a population heterogeneity is usually observed in polluted environments, indicating that heterogeneity is positively related to survival capacity under adverse conditions [23]. Heterogeneity of yeast populations obtained by evolutionary engineering is a commonly observed phenomenon, as discussed previously [22]. Certainly, the harsh conditions that resulted from HSSL composition imposed to *S. stipitis* along 382 generations in CSTR promoted the build-up of a heterogeneous population capable of better withstanding the inhibitors due to the yeast cells diversification actions (for example, detoxification, growth, fermentation). This heterogeneity within POP can then be seen as an advantage during the adaptation process, helping and improving the fermentation process since part of the population could be responsible for the medium detoxification at the expense of energy, allowing the other part to grow and ferment in the same medium.

During all the adaptation process, the ethanol production rate was low, attaining a final value (at 60% HSSL) of 0.32 g.L^−1^.h^−1^ only. This was, however, expected for *S. stipitis* under conditions of full aerobicity [7]. But aerobic conditions were chosen in order to speed up the adaptation process, as yeast cell growth and rapid increase in number of generation were needed for the adaptation. Also, in the shake flasks experiment, the conditions imposed (agitation rate of 180 rpm and a working volume of only 10% of the total Erlenmeyer volume) promoted respiratory metabolism and growth, although oxygen concentration was not measured. All these facts were probably the reason why the difference in ethanol production rate between PAR and the isolates was so noticeable, during the shake flasks evaluation experiments. Adaptation conditions were chosen to allow *S. stipitis* growth in HSSL, so POP’s main priority was to grow in the presence of all inhibitors. We hypothesize that a shift in yeast metabolism was observed, when HSSL started to be fed to the reactor, as both xylose uptake and ethanol production rates started decreasing, whereas glucose uptake rate increased. This could mean that the population was redirecting its metabolism towards growth and ATP generation as a result of the introduction of HSSL inhibitors that increased the energetic needs of the culture. By activating the cells’ survival mechanisms, additional energy supplies must be generated with metabolic production of higher levels of ATP, which would explain why yeast cells redirected the carbon flux from the alcoholic fermentative metabolism to the aerobic growth metabolism, that is a more energetic one [6].

The bioreactors batch tests were performed with the oxygen feed controlled at 0.24 L.min^−1^ and with 60% of HSSL in order to evaluate the differences in bioethanol production between C_4_, PAR and POP at a lower aeration rate. In this experiment, C_4_ achieved an ethanol conversion efficiency of 32%, higher than the one attained by PAR (26%). Bajwa *et al*. were able to achieve a 50% ethanol conversion efficiency in wood hydrolysates with two mutant strains of *S. stipitis* obtained by genome shuffling in a hardwood SSL [24]. Nevertheless, the operational conditions used by Bajwa *et al*. were different than the ones used in this study, a high cell density inoculum was used and the fermentation was performed with poplar hydrolysate. Also, the approach used to obtain the isolates was different but both aimed at the achievement of more robust *S. stipitis* strains. For higher ethanol titers, micro-aerophilic conditions with a tight oxygen control are needed [25], and the required level depends not only on the strain used but also on the fermentation medium. Further studies will now be performed in order to determine the optimal aeration conditions for bioethanol production from undetoxified HSSL by C_4_.

## Conclusions

*S. stipitis* was successfully adapted to 60% (*v*/*v*) undetoxified eucalyptus HSSL. The obtained population showed significant improvements compared with the parental strain, when grown in undetoxified HSSL. A stable isolate, C_4_, with an improved performance in undetoxified HSSL compared to the parental strain was successfully obtained from it. C_4_ achieved higher substrate uptake rates and ethanol conversion efficiency (32%) than the parental strain, thereby representing a major advantage in the bioprocessing of HSSL, which can lead to the industrial application of *S. stipitis* in biorefineries.

## Methods

### HSSL supply and pre-treatment

HSSL from magnesium-based acid sulfite pulping of *E. globulus* was supplied by Caima-Indústria de Celulose SA - ALTRI (Constância, Portugal). Pre-evaporated HSSL was collected and chemically pre-treated by adjusting the pH to 7.0 with KOH pellets, followed by aeration with compressed air for 2 h.L^−1^. The precipitated colloids were removed by centrifugation at 5,000 rpm (4,696 × *g*) during 20 min at 4°C in a Thermo Scientific Heraeus Megafuge 16R centrifuge (Waltham, MA, USA), and the supernatant was filtered using a 1.0-μm glass microfiber filter Fioroni grade 260 (Ingré, France).

### Microorganism and media

*S. stipitis* NRRL Y-7124 (PAR) was gently supplied by the Agricultural Research Service Culture Collection at the National Center for Agricultural Utilization Research, USDA. The yeast culture was grown at 28°C ± 0.5°C, maintained at 4°C on YM agar slants (3.0 g.L^−1^ yeast extract, 3.0 g.L^−1^ malt extract, 5.0 g.L^−1^ peptone) supplemented with 10.0 g.L^−1^ glucose and 20.0 g.L^−1^ agar.

### Pre-cultivation

In all experiments, colonies from YM agar slants were inoculated in a CD medium containing 5.0 g.L^−1^ (NH_4_)_2_SO_4_; 0.5 g.L^−1^ MgSO_4_.7H_2_O, 3.0 g.L^−1^ KH_2_PO_4_, vitamins and trace elements as previously reported [26] and the appropriate sugar concentration. All liquid cultures were performed so that they always contained the same initial concentration of sugars as in HSSL (25 g.L^−1^ xylose and 2.3 g.L^−1^ glucose) [8]. CD medium was buffered at pH 5.5 with 50 mM potassium hydrogen phthalate [27]. Erlenmeyer flasks with a working volume that corresponded to 10% (*v*/*v*) of the total flask volume were used at 180 rpm and 28°C.

### Strain adaptation

A 2-L B. Braun Biotech International Controller micro DCU-300 bioreactor (Melsungen, Germany) with a working volume of 1 L was set up to perform the continuous adaptation of *S. stipitis* to increasing concentrations of HSSL. Stirring was controlled at 300 rpm and temperature set at 28°C. The pH was maintained at 5.5 by addition of 3 M KOH or H_2_SO_4_, and air was supplied at a flux of 0.3 L.min^−1^. Before starting the continuous operation, the reactor was operated in batch mode with CD medium in order to increase biomass concentration. Two separated pumps were used to feed HSSL and CD medium to the reactor so that HSSL concentration could be varied from 0% to 60% (*v*/*v*), and the flux of each pump was calibrated in order to obtain a dilution rate of 0.2 h^−1^. The percentage of HSSL was increased after the cells had reached a steady state. The reactor was operated for 382 generations with increasing HSSL percentage until 60% (*v*/*v*). Samples were taken every day for OD_620nm_ in a Shimadzu UV-Mini 1240 spectrophotometer (Kyoto, Japan) and high-pressure liquid chromatography (HPLC) analysis. The final population (POP) obtained in the CSTR was stored at −80°C in 20% (*v*/*v*) glycerol solution.

### Isolation and characterization of POP isolates

POP cells were plated in agar slants containing 40% YM supplemented with 10 g.L^−1^ xylose (YM_xyl_) and 60% HSSL (*v*/*v*). Ten colonies were selected randomly from the fastest grown colonies and individually streaked in non-selective YM_xyl_ medium. The isolates were designated from C_1_ to C_10_ and each one re-streaked ten times in YM_xyl_. For the isolates characterization in HSSL, cells were inoculated in 100 mL Erlenmeyers with 10 mL of CD medium containing 60% (*v*/*v*) of HSSL and the growth of the ten isolates together with PAR and POP in HSSL was assessed by measuring OD_620nm_ overtime. Samples were taken at the beginning and the end of each fermentation, for HPLC analysis. Biological duplicates were performed. After this screening, the clone showing the most effective growth was chosen to be further characterized and compared with PAR and POP in batch reactor tests.

### Batch reactor tests

Batch tests to compare the ability of producing bioethanol from undetoxified HSSL of C_4_, POP and PAR were carried out in a 1-L B. Braun BioLab reactor (Melsungen, Germany), using 800 mL of working volume and automatic control to operate at 28°C with 240 rpm, an aeration feeding of 0.24 L.min^−1^ and pH 5.5 by addition of 3 M KOH or H_2_SO_4_. Tests were performed with CD medium containing 60% of HSSL (*v*/*v*). Samples were taken over time for OD_620nm_ and HPLC analysis and at the end of each test for dry weight and CFU determinations. Each batch reactor corresponded to a single experiment.

### Analytical methods

Cell dry weight was determined at the end of the fermentations. Five millilitres of samples were filtered with 0.45-μm pore diameter membranes from Whatman ME 25/21 ST (Maidstone, England) and washed with 15 mL of distilled water. Each filter was then dried at 100°C until constant weight. Determinations were performed in triplicate.

CFUs were determined at the end of each fermentation by performing serial dilutions of 500 μL of sample into 4.50 mL of sterile 0.9% NaCl. One hundred microliters of the selected dilutions were then plated in YM solid medium and incubated for 48 h at 28°C.

Glucose, xylose, acetic acid and ethanol were analysed by VWR-Hitachi LaChrom Elite® HPLC system (Tokyo, Japan) using a 10-μm EurokatH from Knauer (Berlin, Germany) ion exchange column, 300 × 7.5 mm with an Gecko 2000 oven (Hattersheim, Germany) set at 40°C and refraction index detector Hitachi RI Detector L-2490 (Tokyo, Japan). The eluent was sulphuric acid 0.01 N, with a flow rate of 0.4 mL.min^−1^. All samples were centrifuged and filtered off with 0.20-μm filters CoStar Spin-X (Corning, NY, USA) before the analysis. A standard calibration curve was obtained by injecting standards and used for all analysed compounds.

### Calculations of substrate consumption and product formation rates

In the shake flasks experiments, volumetric uptake or production rates were calculated by the average between the consumed compounds or produced compound values over time from two biological duplicates. For the batch reactor tests, volumetric uptake and production rates were determined at the end of the batch (average rates) or at the maximum ethanol concentration (maximum rates) that were evaluated from the sampling over time.

## Abbreviations

2G: second generation
CD: chemically defined
CFU: colony-forming units
COD: chemical oxygen demand
CSTR: continuous-stirred tank reactor
HPLC: high-pressure liquid chromatography
HSSL: hardwood spent sulfite liquor
OD: optical density
SSLs: spent sulfite liquors
